# Genomic insights into the diversity, antibiotic resistance, and virulence potential of staphylococci isolated from pediatric patients with chronic otitis media with effusion (COME)

**DOI:** 10.7717/peerj.20782

**Published:** 2026-03-24

**Authors:** Jonald Low, Haohong Tu, Mohamed Elbadawey, Ali Wayes, Nicholas Jakubovics, Siew Woh Choo, Wee Wei Yee

**Affiliations:** 1 School of Science, Monash University Malaysia, Selangor, Malaysia; 2 Department of Biology, Wenzhou-Kean University, Zhejiang, China; 3 Tanta University, Gharbia Governorate, Egypt; 4 Kirkuk University, Kirkuk, Iraq; 5 Newcastle University, Newcastle upon Tyne, United Kingdom; 6 Zhejiang Bioinformatics International Science and Technology Cooperation Center, Zhejiang, China; 7 Wenzhou Municipal Key Laboratory for Applied Biomedical and Phameceutical Informatics, Zhejiang, China

**Keywords:** Coagulase positive *Staphylococcus*, Coagulase negative *Staphylococcus*, Chronic otitis media with effusion, Antibiotic resistance, Virulence factor, Horizontal gene transfer

## Abstract

**Background:**

Chronic otitis media with effusion (COME) is a prevalent pediatric condition characterized by persistent middle ear effusion, potentially leading to hearing loss and developmental delays.

**Methods:**

We investigated the diversity, antibiotic resistance, and virulence potential of *Staphylococcus* species in COME through whole genome sequencing of 16 clinically-derived strains isolated from pediatric patients in the United Kingdom. *De novo* genome assembly and annotation were performed on Illumina reads. Phylogenetic analyses using 16s rRNA gene, multilocus sequence typing (MLST) and core genome single nucleotide proteins (SNPs) elucidated evolutionary relationships. Species identification was confirmed through *in silico* DNA-DNA hybridization (ANIb and GGDC). Resistance genes were detected using AMRFinderPlus and Comprehensive Antibiotic Resistance Database (CARD), and virulence factors were identified using VFanalyzer. Pangenome analysis identified unique species-specific genes.

**Results:**

Phylogenetic analysis revealed three coagulase positive *Staphylococcus* (CoPS) and 13 coagulase negative *Staphylococcus* (CoNS), with identification of a potential new *S. aureus* subspecies (strain NU84) Twenty-four genes conferred resistance to nine antibiotic classes, particularly beta-lactams commonly used for COME treatment. Notably, all 16 strains harbored *blaTEM-116* and *aph(3’)-IIa* genes, typically associated with gram-negative bacteria and previously unreported in human *Staphylococcus* isolates, suggesting horizontal gene transfer from Enterobacteriaceae. CoPS strains exhibited higher acute virulence potential contributing to COME onset, whereas CoNS, particularly *S. epidermidis,* harbored genes promoting persistence through immune evasion and biofilm formation, consistent with the chronic nature of COME.

**Conclusion:**

Our genomic analysis shows that COME-associated *Staphylococcus* species have significant pathogenic potential due to acquired resistance and virulence genes. The discovery of gram-negative resistance genes in all *Staphylococcus* strains indicates horizontal gene transfer may enhance pathogenicity. These findings highlight the urgent need for surveillance and targeted therapies against emerging multidrug-resistant strains in COME treatment.

## Introduction

Chronic otitis media with effusion (COME), a chronic inflammatory disorder of the middle ear without acute inflammation symptoms, is characterized by the persistence of non-purulent middle ear effusion fluid (MEEF) in the tympanic cavity for more than three months (Bhutta et al., 2017; Vanneste & Page, 2019). As one of the most common childhood diseases worldwide, COME often remains untreated due to its asymptomatic nature, leading to conductive hearing loss and consequent complications such as delayed speech development, abnormal behavior, poor social skills, and reduced academic achievement (Olencewicz, Holt & Sharma, 2025; Rosenfeld et al., 2016).

The pathogenesis of COME is multifactorial, influenced by individual and environmental factors (Blanc et al., 2018). Individual risk factors include genetic predisposition, laryngopharyngeal and gastroesophageal reflux diseases, eustachian tube dysfunction, immunological disorders, and craniofacial development defects (Assiri, Hudise & Obeid, 2024; O’Reilly et al., 2015). Environmental factors, such as bacterial infection and exposure to tobacco smoke, also contribute to COME development (Assiri, Hudise & Obeid, 2024; Cheng et al., 2017).

Recent genomic studies on COME have revealed a diverse array of bacterial populations, with *Haemophilus influenzae*, *Alloiococcus otitidis*, *Streptococcus* and *Staphylococcus* species being dominant (Ngo et al., 2016; Chan et al., 2016; Fekete et al., 2024). While these studies provided important insights into microbial diversity compared with traditional culture-based analyses, they were largely limited to taxonomic profiling. Our study extends beyond prior genomic investigations by examining genomic diversity underlying key clinical features that directly contribute to COME severity such as antimicrobial resistance, biofilm formation, and virulence. Among the suspected pathogens, *Staphylococcus* species are increasing implicated in COME etiology due to their strong biofilm-forming abilities (Locke et al., 2022). As a key factor in chronic infection and antibiotic resistance, biofilm formation has been identified in approximately 92% of middle ear mucosa of COME patients, and on the surface of ventilation tubes recovered from COME-affected children (Vestby et al., 2020; Gil-Perotin et al., 2012).

Staphylococci, classified into coagulase-positive (CoPS) and coagulase-negative (CoNS) groups, are common gram-positive bacteria colonizing mucous membranes and skin (Liu et al., 2024). The CoPS representative, *Staphylococcus aureus*, particularly methicillin-resistant strains (MRSA), is a leading cause of nosocomial disease and is increasingly resistant to multiple antibiotics (Kaur & Chate, 2015) CoNS, the most frequently found group in MEEF from COME patients (Min et al., 2019; Kim et al., 2017), are increasingly considered causative factors in bacterial infections due to their biofilm formation ability, which may contribute to the ineffectiveness of antibiotic treatment in COME (Sharma, Misba & Khan, 2019).

This study aims to conduct a detailed genomic analysis of *Staphylococcus* strains from COME patients, focusing on identifying resistance genes and potential new subspecies, and linking these genomic insights to clinical outcomes to provide a foundation for developing targeted treatments and advancing personalized medical approaches. To achieve these goals, we sequenced 16 new clinically-derived *Staphylococcus* strains isolated from COME patients in the United Kingdom using high-throughput Illumina HiSeq X10 technology and performed comprehensive bioinformatics analyses.

## Materials and Methods

Ethical approval for this study was granted by the NHS Research Ethics Service Committee (North East Newcastle and North Tyneside), approval number 15/NE/0225. Written informed consent was obtained from all patients and/or carers participate in the study. Samples were collected as part of routine surgery.

### Sample collection and processing

MEEF samples were collected from teenagers under 16 years old who were diagnosed with COME and had been experiencing persistent symptomatic COME for at least three months. The recruitment of patients was done between November 2015 and December 2016 at the Freeman and Royal Victoria Infirmary Hospitals, Newcastle upon Tyne, UK. Patients with recurrent AOM, congenital craniofacial malformations or previous history of grommet insertion, myringotomy were excluded from the study. Firstly, wax and other debris were atraumatically cleaned out from the ear canal according to standard aseptic technique. A small surgical incision was made in the eardrum to aspirate MEEF using a sterile suction catheter through myringotomy. Stringent precautions were taken to prevent any contact with the external ear canal during the procedure. Aspirated MEEF was carefully collected into a sterile mucous trap containing two ml of 0.9% saline and transported on ice to the Oral Microbiology Laboratory, Newcastle University for immediate processing.

### Isolation and of staphylococci

To isolate a range of microorganisms from MEEF, samples were split into three equal portions and 50 µL were plated in duplicate on three different types of medium: (i) Blood Agar composed of (per L) 37 g Brain Heart Infusion (Melford Laboratories, Suffolk, UK), five g yeast extract (Melford) and 15 g agar granules (Melford), autoclaved and cooled, then supplemented with 5% (v/v) defibrinated horse blood (TCS Biosciences, Buckingham, UK), (ii) Chocolate Agar containing the same ingredients as Blood Agar, with an extra step of heating at 75–80 °C for 10 min after the addition of horse blood, and (iii) Fastidious Anaerobe Agar (FAA), prepared by incubating 46 g of Fastidious Anaerobe Broth (Lab-M, Lancashire, UK) with 15 g agar (Melford), autoclaving, cooling, and adding 5% (v/v) defibrinated horse blood. Media were autoclaved before any addition of blood at 121 °C for 20 min and allowed to cool at 45–50 °C. After plating samples, Blood and Chocolate Agar were incubated at 37 °C and 5% CO_2_ for up to 7 days and FAA plates were incubated anaerobically in a BugBox (Baker Ruskinn, Bridgend, UK) in 10% CO_2_, 10% H_2_ and 80% N_2_ for up to 14 days. Plates were checked periodically and colonies were picked and subcultured three times to establish pure cultures. Isolated colonies were suspended in 20 mL BHYE broth (37 g/L Brain Heart Infusion and 5 g/L yeast extract) and incubated for 24–48 h at 37 °C and 5% CO_2_. Cultures were checked for contamination under a light microscope (Leica DM 750) prior to centrifugation (3,600 g in a swing out rotor for 10 min at 4 °C). The pellet was suspended in one ml of BHYE medium and diluted by the addition of one volume of glycerol (Sigma Aldrich, UK) before being stored at −80 °C.

### Preliminary identification of isolates

During initial isolation, preliminary identification was performed based on light microscopy, Gram staining, colony morphology and growth in aerobic/anaerobic conditions. Screened isolates were then identified to species level using MALDI-TOF mass spectrometry on a Bruker Daltonik MALDI Biotyper (Bruker UK Ltd, Coventry, UK) at the Freeman Hospital, Newcastle upon Tyne, UK.

### Genomic DNA extraction

A total of 20 mL of *Staphylococcus* strains were cultured in Tryptic Soy Broth (TSB, Melford) and incubated overnight (37 °C with shaking at 200 rpm). Supernatant was removed after centrifuging the strains (3,600g for 10 min in a swing out rotor). Pellets were resuspended with 150 µL of pre-warmed spheroplasting buffer (20 mM Tris–HCl, pH 6.8; 10 mM MgCl_2_; 26% w/v raffinose.5H_2_O) supplemented with a final concentration of 250 µg/mL lysozyme (Sigma Aldrich, St. Louis, MO, USA) and 5 µg mutanolysin (Sigma Aldrich, St. Louis, MO, USA; reconstituted at 10,000 U/mL), before being incubated at 37 °C for 30 min. Next, 150 µL of 2x T&C Lysis Solution (Epicenter) was added along with 25–50 mg of acid-washed glass beads (0.1 mm) and cells were disrupted in a bead-lysis machine (Qiagen Tissue Lyser Ltd., Qiagen, Hilden, Germany) for five minutes at 50 Hz. MasterPure™ Gram Positive DNA Purification Kit (Epicentre**^®^** Biotechnologies, Madison, WI, USA) was then utilized for DNA extraction following the manufacturer’s instructions. Extracted DNA was suspended in the elution buffer (25 µL 10 mM Tris pH 8.5) and stored at −20 °C for further use. The concentration of extracted DNA was measured using NanoDrop ND-1000 Spectrophotometer (Thermo Scientific, Waltham, MA, USA) with DNA-50 setting.

### Library preparation and whole-genome sequencing

Genomic DNA extracted was randomly fragmented and quality checked using FastQC. The DNA fragments were end-repaired using T4 DNA polymerase, Klenow fragment and T4 polynucleotide kinase followed by the ligation of adapter sequences. DNA fragments were electrophoresed for size-selection before undergoing amplification using polymerase chain reaction. DNA sequencing was performed on the Illumina HiSeq X10 platform with PE150 mode. Quality control was done on the Illumina reads using FastQC and Trimmomatic 0.36 was utilized to trim the adapter sequences (Andrew, 2010; Bolger, Lohse & Usadel, 2014). Duplicated reads and the reads with at least 40% of low-quality bases (Phred quality score≤ 20) or 40% of ambiguous nucleotides were filtered out to generate high quality read data for genome assembly. Negative controls were not included as environmental contamination during whole-genome sequencing from isolated bacteria is rare and easily filtered bioinformatically.

Notably, our genomic sequencing approach is uniquely suited to address the goals of the study because it: (1) comprehensively identifies resistance genes missed by traditional methods, (2) detects horizontal gene transfer events critical for understanding pathogen evolution in COME, and (3) provides resolution to characterize previously undetected resistance mechanisms in *Staphylococcus* species. This directly addresses current surveillance limitations where conventional methods fail to capture the full extent of antimicrobial resistance.

### Genome assembly and annotation

Sequencing reads were *de novo* assembled using SPAdes and Qiagen CLC Genomic Workbench respectively (Bankevich et al., 2012; QIAGEN, 2022). Different Kmer sizes (21, 42, 63) were tested for the genome assemblies. Contig number, N50 size, minimum contig size and genome sizes of the assembled genomes were identified using the Python script “assembly_stats.py” from Github (Nicholas, 2014). The selection of the best assembled genome was determined according to the genome size (bp), GC content (%), N50 size, contig number and minimum contig size. All assembled genomes were annotated using the Rapid Annotation Subsystem Technology (RAST) pipeline, which is a fully automated annotation pipeline for complete or draft archaeal and bacterial genomes (Aziz et al., 2008).

### Phylogenetic tree construction

For the construction of phylogenetic trees, the sequences of *Staphylococcus* strains of different species were included to determine the taxonomic relationships among these staphylococci with our strains (Table S2). Three phylogenetic trees were reconstructed using MEGA11 (Tamura, Stecher & Kumar, 2021). After having all the specific sequences of *Staphylococcus* strains as input, CLUSTALW was used to perform multiple sequence alignment followed by phylogenetic tree reconstruction using different methods.

For the single gene approach, we used 16S rRNA gene sequences. For the multiple gene approach, we used seven commonly employed housekeeping genes: *aroE* (Skikimate dehydrogenase), *carA* (Carbomyl phosphate synthetase A), *glpF* (Glycerol uptake facilitator protein), *gmk* (Guanylate kinase), *pta* (Phosphate acetyltransferase), *tpi* (triosephosphate isomerase) and *yqiL* (Acetyl coenzyme A acetyltransferase) in order to reconstruct more robust trees. Both of these phylogenetic trees were inferred using the maximum-likelihood algorithm with 1000 bootstrap replicates. For the core-genome SNP approach, all genome sequences were uploaded to the Panseq (Pan-Genome Sequence Analysis Program) (Laing et al., 2010). Panseq aligned all sequences and identified the highly considered genomic regions (or core genome) with the parameters of 90 percent Identity Cutoff and 16 core Genome Threshold (ie present in 100% of genomes). The core-genome SNPs were identified and used to generate a phylogenetic tree. This phylogenetic tree was constructed using MEGA11 with the neighbor-joining algorithm and nodal support was estimated using 1,000 replicates.

### *In silico* DNA-DNA hybridization

To evaluate the genetic relatedness between the genomes of our strains and their closely related species, the genome-based taxonomic analyses were performed using both average nucleotide identity based on BLAST algorithm (ANIb) and genome-to-genome distance calculation (GGDC) (Richter et al., 2016; Meier-Kolthoff et al., 2022). Genetic relatedness between *Staphylococcus* species was determined by calculating the average nucleotide identity (ANI) between DNA regions of high sequence similarity based on BLAST + using JSpeciesWS software (Richter et al., 2016). ANIb values greater than 95% are used as a threshold for species delineation in prokaryotes (Richter & Rossello-Mora, 2009). GGDC was performed using Genome-to-Genome Distance Calculator web server (Meier-Kolthoff et al., 2022). Results generated through Formula 2 (identities/HSP length) were used as it provides more accurate and reliable estimates of digital DNA-DNA hybridization score (dDDH) which has higher correlation with the result of wet-lab DDH experiments than other formulas. *Staphylococcus* strains showing dDDH value of above 70% indicates the same species, while above 79% indicates the same subspecies (Dekio et al., 2021; Meier-Kolthoff et al., 2014). The reference genomes used for each sample in both GGDC and ANIb were based on the nearest *Staphylococcus* strains retrieved from the core genome SNPs phylogenetic tree. Once we obtained the results, a similarity matrix was created with all pairwise genomic comparisons and further used to generate heatmap using the Pheatmap and ggplot2 packages in R (Rstudio Team, 2022).

### Identification of putative virulence genes and resistance genes

To identify putative virulence genes in the assembled genomes of *Staphylococcus* strains, we searched these genome sequences against the Virulence Factor Database (VFDB) using VFanalyzer (Liu et al., 2019). *In silico* screening of the assembled genomes for the presence of resistance genes was performed through NCBI Antimicrobial Resistance Gene Finder (AMRFinderPlus) database and Comprehensive Antibiotic Resistance Database (CARD), which was preferably for mutations conferring resistance genes (Feldgarden et al., 2021; McArthur et al., 2013). Genomic islands of the *Staphylococcus* strains were predicted using Islandviewer to identify horizontally acquired DNA segments contributing to antibiotic resistance and virulence (Bertelli, Laird & Williams, 2017).

### Pan-genome analysis/gene family clustering

Pan-genome analysis was performed using the sequences from our strains. The gff3 annotation files were obtained through Prokka and used as an input of Roary to generate gene_presence_absence.csv file (Seeman, 2014). All genes from staphylococcal genome were clustered by Roary using 75% BLASTP similarity, where genes with at least 75% protein identity > 90% length are considered orthologous (Page et al., 2015). This threshold produces stable, biologically meaningful clusters and balance sensitivity to within-species diversity with accurate clustering in bacteria with high genomic plasticity (Socarras et al., 2025; Bohr, Mortimer & Pepperell, 2020). Besides, the core and accessory genomes were identified by the pan-genome pie chart generated through Roary. The same procedures were repeated to compare *S. aureus* between our samples with other published environmental strains used in this analysis. Functional annotations were performed on *S. aureus* genes within the core genome and accessory genome using RAST web server, respectively (Aziz et al., 2008).

## Results

### Patient recruitment and strain isolation

Thirty-nine patients were recruited: 34 for myringotomy and grommet insertion (COME cases) and five for adenotonsillectomy (controls without COME). From 34 COME patients, 59 MEEF samples yielded 79 bacterial strains after isolation and culture. Twenty-seven strains were identified as *Staphylococcus* spp. by MALDI-TOF, with 16 selected for whole-genome sequencing to represent phylogenetic diversity.

### Genome summary

We generated 62.5 million raw reads for 16 *Staphylococcus* strains isolated from COME patients in the UK (Table S1). The assembled genomes had sizes ranging from 2.2 to 2.8 Mb, with an average GC content of 33.1% (Table 1). The number of predicted protein-coding genes (CDS) and tRNAs ranged from 2,220 to 3,067 and 43 to 60, respectively. Strain NU72 had the smallest genome size (2.24 Mb) and the lowest number of CDS (2,220), while strain NU68 had the largest number of CDS (3,067) with a genome size of 2.61 Mb. The variation in CDS numbers may be attributed to species-level differences accumulated over evolutionary time.

**Table 1:** Genome statistics and species identities. The table shows the number of contigs, protein coding genes (CDSs), RNA, GC content, and Virulence, Disease & Defence Genes (VDDG) of *Staphylococcus* strains.

Strain	NU93	NU92	NU91	NU85	NU84	NU83	NU72	NU71
Genome size (bp)	2,497,245	2,435,504	2,796,976	2,699,909	2,706,210	2,270,796	2,242,180	2,537,359
N50 (Kbp)	74.6	1,241,533	125.7	338.5	712.7	556.6	219.3	125.7
Contig No.	84	55	73	60	21	27	54	49
% of GC	32.0	32.8	32.8	32.4	32.8	37.1	31.3	31.9
CDS No.	2,401	2,335	2,674	2,648	2,553	2,247	2,220	2,454
VDDG No.	55	47	70	48	58	25	44	48
tRNA No.	57	59	44	49	47	49	56	57
rRNA No.	4	9	4	4	3	4	4	5
Species	*S. epidermidis*	*S. aureus*	*S. aureus*	*S. caprae*	*S. aureus*	*S. auricularis*	*S. hominis*	*S. epidermidis*
Strain	NU65	NU64	NU58	NU57	NU56	NU41	NU70	NU68
Genome size (bp)	2,807,938	2,590,674	2,503,076	2,656,843	2,469,963	2,661,562	2,458,398	2,613,534
N50 (Kbp)	151.7	226.9	119.3	415.0	121.0	135.8	116.6	116.8
Contig No.	42	63	44	194	56	63	50	68
% of GC	32.6	31.4	32.1	32.8	32.0	31.9	38.7	36.0
CDS No.	2,675	2,523	2,384	2,726	2,374	2,633	2,633	3,067
VDDG No.	60	54	57	56	56	52	52	66
tRNA No.	43	51	58	59	57	56	48	60
rRNA No.	3	5	5	5	5	5	4	9
Species	*S. aureus*	*S. pasteuri*	*S. epidermidis*	*S. aureus*	*S. epidermidis*	*S. epidermidis*	*S. pettenkoferi*	*S. simulans*

### Phylogenetic analysis and subspecies identification

To determine the phylogenetic relationships of our 16 *Staphylococcus* strains, we reconstructed phylogenetic trees by incorporating the sequences of nearly all recognized *Staphylococcus* taxa and our strains (Table S2). Phylogenetic trees reconstructed using single gene (*16S rRNA*), multiple genes (*aroE*, *carA*, *glpF*, *gmk*, *pta*, *tpi*, *yqiL*), and core-genome SNP approaches consistently supported the taxonomic positions of the 16 *Staphylococcus* strains (Figs. S1–S3). There are 1,784 core-genome SNPs generated from PanSeq which have a total length of 1.4 M base pairs.

**Figure 1:**
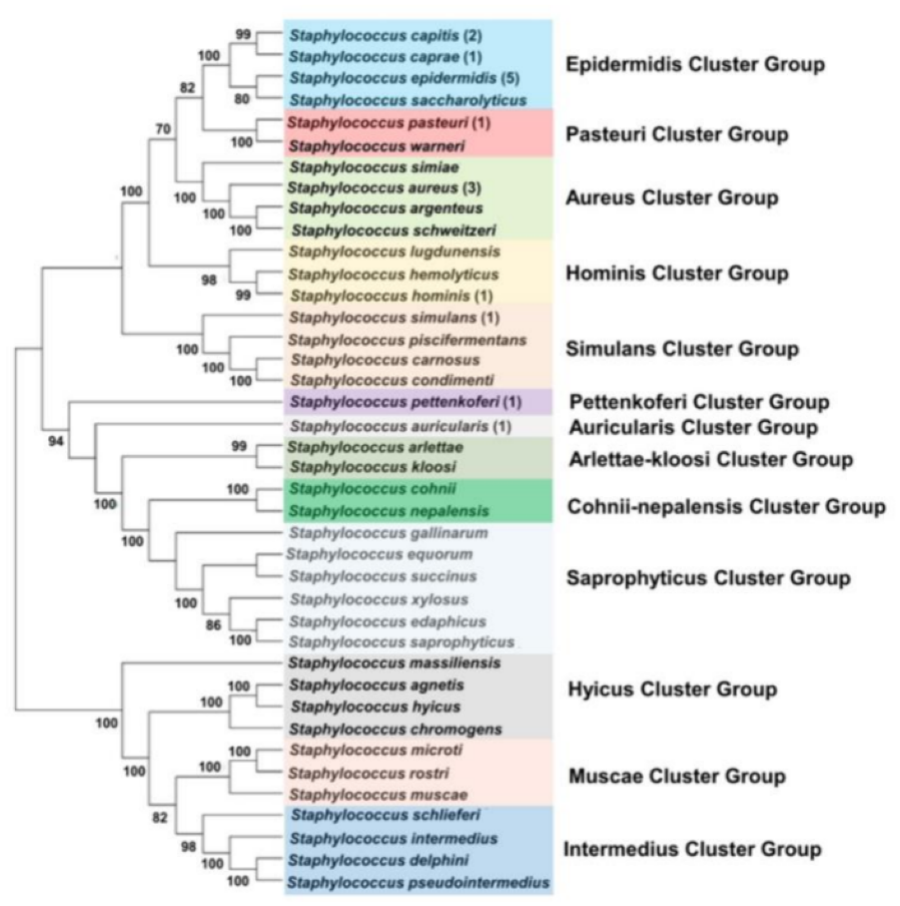
*Staphylococcal* species were distributed into 13 cluster groups according to the phylogenetic clades based on branching patterns. A summary phylogram adapted from the staphylococcal phylogeny estimated from concatenated analyses which excludes out our *Staphylococcus* strains. The number besides species name indicated the times of that particular species having closest phylogenetic relationships with our *Staphylococcus* strains. Bootstrap consensus trees were inferred from 1,000 replicates. Bootstrap values of greater than 70 are shown.

We distributed the *Staphylococcus* species into 13 clusters according to the staphylococcal phylogeny estimated from concatenated analyses (Fig. 1). Each cluster group was named based on the branching pattern and kept as consistent as possible with the original nomenclature put forth by the previous study (Lamers et al., 2012). Generally, our findings are in broad agreement with gene tree-based reports of staphylococcal phylogeny.

**Figure 2:**
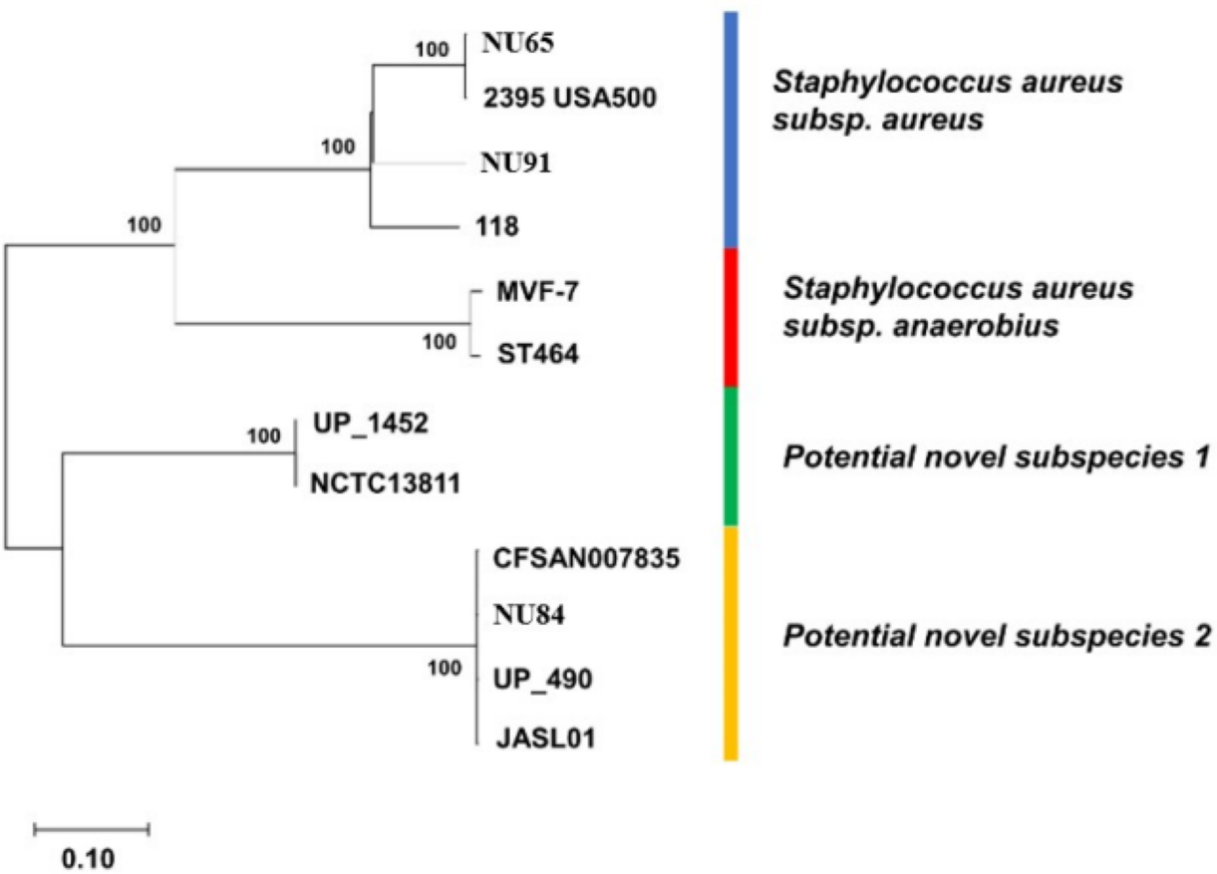
The core-genome SNP-based phylogenetic tree for *S. aureus* strains. Our data showed four phylogenetic clades which are *S. aureus subsp. aureus*, *S. aureus subsp. anaerobius* and the remaining are potential novel subspecies. Bootstrap values of greater than 70 are shown.

In silico DNA-DNA hybridization (ANIb and GGDC) analyses further confirmed the strains’ identities (Table 1, Figs. S4–S5), revealing three *S. aureus* (CoPS) and 13 CoNS strains (five *S. epidermidis*, two *S. capitis*, one *S. caprae*, one *S. hominis*, one *S. auricularis*, one *S. pasteuri*, one *S. pettenkoferi*, and one *S. simulans*). Genome-to-Genome Distance Calculator (GGDC) analysis identified four *S. aureus* subspecies, with NU91 and NU65 belonging to *S. aureus* subsp. *aureus*, while NU84 and two reference strains (UP_1452 and NCTC13811) potentially represent novel subspecies (Fig. 2, Fig. S8) based on their dDDH values between 70% to 79%. Moreover, strain NU72 was identified as *S. hominis* subsp. *novobiosepticus* (Figs. S6–S7). *S. aureus* subspecies are distinguished by catalase production, while *S. hominis* subspecies are differentiated by novobiocin resistance and aerobic acid production from N-acetylglucosamine and D-trehalose (Bauer et al., 2025; Szemraj et al., 2025).

### Resistance gene profiles

AMRFinderPlus and CARD identified 24 putative resistance genes in the *Staphylococcus* genomes, conferring resistance to nine antibiotic classes (Table 2, Fig. S9) (Feldgarden et al., 2021; McArthur et al., 2013). Among of these classes are aminoglycoside, beta-lactam, trimethoprim, fosfomycin, fusidic acid, MLS_b_ (macrolide, lincosamide and streptogramin), tetracycline, and multidrug efflux pump. The same *Staphylococcus* species generally possess similar resistance gene profiles, whereas different species may have different profiles. For example, *S. aureus* harbors additional resistance genes encoding multidrug efflux pumps such as *mepA* and *mepR,* whereas *S. epidermidis* exhibits extra resistance to tetracycline through the *tetA* gene.

**Table 2:** Distribution of resistance genes in *Staphylococcus* strains isolated from COME patients into nine antibiotic classes.

Antibiotic class	Resistance gene	*S. aureus*	*S. epidermidis*	*S. capitis*	*S. auricularis*	*S. hominis*	*S. caprae*	*S. pasteuri*	*S. pettenkoferi*	*S. simulans*
Aminoglycoside	*ANT(4′)-Ia*	0	1/5^ˆ^	0	0	0	0	0	0	0
	*ANT(9)-Ia*	1/3^ˆ^	0	0	0	0	0	0	0	1/1^*^
	*aph(3′)-IIa*	3/3^*^	5/5^*^	2/2^*^	1/1^*^	1/1^*^	1/1^*^	1/1^*^	1/1^*^	1/1^*^
Beta-lactams	*blaI*	3/3^*^	4/5^ˆ^	2/2^*^	0	1/1^*^	0	1/1^*^	0	0
	*blaR1*	3/3^*^	1/5^ˆ^	1/2^ˆ^	0	0	0	1/1^*^	0	0
	*blaZ*	3/3^*^	4/5^ˆ^	2/2^*^	0	1/1^*^	1/1^*^	1/1^*^	0	0
	*blaTEM- 116*	3/3^*^	5/5^*^	2/2^*^	1/1^*^	1/1^*^	1/1^*^	1/1^*^	1/1^*^	1/1^*^
	*mecA*	1/3^ˆ^	2/5^ˆ^	0	0	0	0	0	0	0
Fosfomycin	*fosB*	2/3^ˆ^	5/5^*^	1/2^ˆ^	0	0	0	1/1^*^	0	0
Fusidic acid	*fusB*	0	3/5^ˆ^	2/2^*^	0	0	0	0	0	0
	*fusC*	0	0	0	0	1/1^*^	0	0	0	0
Macrolide	*ermA*	1/3^ˆ^	0	0	0	0	0	0	0	1/1^*^
	*ermC*	1/3^ˆ^	0	0	0	0	0	0	0	0
	*mphC*	0	5/5^*^	1/2^ˆ^	0	1/1^*^	1/1^*^	0	0	0
	*msrA*	0	5/5^*^	0	0	1/1^*^	0	0	0	0
Tetracycline	*tetA*	0	5/5^*^	0	0	0	0	0	0	0
	*tet(38)*	3/3^*^	0	0	0	0	0	0	0	0
Trimethoprim	*dfrC*	0	4/5^ˆ^	0	0	0	1/1^*^	0	0	0
Multidrug efflux pump	*norA*	3/3^*^	5/5^*^	2/2^*^	0	0	1/1^*^	1/1^*^	0	0
	*qacA*	0	2/5^ˆ^	1/2^ˆ^	0	0	0	0	0	0
	*qacB*	0	1/5^ˆ^	1/2^ˆ^	0	0	0	0	0	0
	*qacC*	0	2/5^ˆ^	0	0	0	1/1^*^	0	1/1^*^	1/1^*^
	*mepA*	3/3^*^	0	0	0	0	0	0	0	0
	*mepR*	3/3^*^	0	0	0	0	0	0	0	0

**Notes.**

ˆ Present in partial strains of a specific species.

* Present in all strains of a specific species. The number indicates the number of species containing the resistance gene.

Two known mechanisms confer resistance to beta-lactam antibiotics in *Staphylococcus* species: the production of beta-lactamase, encoded by the *blaZ* gene, which hydrolyzes the beta-lactam ring (Gan et al., 2021), and the production of altered penicillin-binding protein, PBP2a, conferred by the *mecA* gene (Lade & Kim, 2023). The *blaZ* gene was present in 12/16 strains, suggesting potential resistance to beta-lactam antibiotics. Three strains (two *S. epidermidis* and one *S. aureus*) harbored the *mecA* gene, producing PBP2a which crosslink peptidoglycan in bacterial cell walls thereby possess lower affinity to beta-lactam antibiotics (Lade & Kim, 2023). Surprisingly, the *blaTEM-116* gene, an extended-spectrum beta-lactamase (ESBL) gene typically found in gram-negative bacteria, and the *aph(3′)-IIa* gene, a transposon-encoded aminoglycoside phosphotransferase usually associated with Enterobacteriaceae, were present in all 16 strains.

Aminoglycosides, effective antibiotics that bind to bacterial 30S ribosomal subunits and interrupt RNA translation (Mahdiyoun et al., 2016), are inactivated by aminoglycoside phosphotransferases produced by *Staphylococcus* species as a self-defense mechanism (Brdová, Ruml & Viktorová, 2024). The *ANT(4′)-Ia* and *ANT(9)-Ia* genes were found sporadically in *S. aureus*, *S. epidermidis*, and *S. simulans*. Modifications in dihydrofolate reductase, encoded by *dfr* genes, confer resistance to trimethoprim, which inhibits tetrahydrofolate production necessary for purine and pyrimidine synthesis (Krucinska et al., 2022). The *dfrC* gene, a chromosome-encoded dihydrofolate reductase, was found in *S. epidermidis* and *S. caprae*, consistent with previous studies showing a high percentage of the *dfrC* gene in *S. epidermidis* among other CoNS (Cave et al., 2019).

Fosfomycin resistance, conferred by the *fosB* gene through enzymatic drug inactivation was observed in *S. epidermidis* and most *S. aureus* strains (Osada et al., 2022). Fusidic acid resistance, mediated by the *fusB* and *fusC* genes encoding an elongation factor G (EF-G) binding protein which inhibits bacterial protein elongation step was detected in *S. hominis*, *S. capitis*, and the majority of *S. epidermidis* strains (González-López et al., 2025). The MLS_b_ antibiotic class comprises several antibiotics such as macrolide, lincosamide and streptogramin which have similar functions of inhibiting bacterial protein synthesis although they are structurally different (Li et al., 2015). MLSb resistance, associated with genes encoding phosphotransferases (*mphC*), ATP-dependent efflux pumps (*msrA*), and ribosome methylases (*erm*), was more prevalent in CoNS, particularly *S. epidermidis*, compared to CoPS (Li et al., 2015). The gene *norA* was the most prevalent resistance gene encoding a multidrug efflux pump in *S. aureus* isolates.

### Comparative pathogenomic analysis

To better understand the pathogenic potential of these staphylococci, we performed a comparative pathogenomic analysis using all genome sequences of *Staphylococcus* strains. VFanalyzer identified 56 putative virulence genes across four functional categories: biofilm-related proteins, immune evasion proteins, enzymes, and toxins (Table S3–S6) (Liu et al., 2019). These genes will be discussed in details below:

### Biofilm-related genes

We identified a suite of 17 genes implicated in the biofilm formation of *Staphylococcus* spp. Key among these are microbial surface components recognizing adhesive matrix molecules (MSCRAMMs) (Ghasemian et al., 2015; Foster et al., 2020). MSCRAMMs are vital for the pathogenesis of staphylococcal infections, facilitating colonization, invasion, and biofilm formation. Predominantly, 13 genes encode MSCRAMMs, including autolysin (*atl*), accumulation-associated protein (*aap*), biofilm associated surface protein (*bap*), fibrinogen-binding protein (*fib*), fibronectin-binding proteins (*fnbA* & *fnbB*), cell wall associated fibronectin binding protein (*ebh*), extracellular matrix binding protein (*emp*), clumping factors (*clfA* & *clfB*), laminin-binding protein (*eno*), collagen binding protein (*cna*) and elastin-binding protein (*ebp*) (Geoghegan & Foster, 2017). Furthermore, the polysaccharide intercellular adhesins (PIA), crucial extracellular polymeric substances in staphylococcal biofilms, are encoded by the *icaABCD* genes (Arciola et al., 2015).

Our analysis reveals frequent occurrences of MSCRAMM genes in clinical *Staphylococcus* strains (Table S3). Notably, the *atl* gene appears in 12 out of 13 strains, consistent with its noted prevalence in biofilm-related research (Fergestad et al., 2021). Similarly, the *ebp* gene is present in 11 out of 13 strains. Our findings align with earlier studies showing an absence of the *bap* gene and the presence of *icaABCD* genes in all *S. aureus* strains examined, mirroring the distribution patterns reported in other *Staphylococcus* species (Chen et al., 2020). Our results also indicate that the *clfB*, *ebh*, *emp*, and *efb* genes are universally present in coagulase-positive staphylococci (CoPS), but absent in coagulase-negative staphylococci (CoNS). Species-specific biofilm-related genes include *fnbA* and *fnbB* in *S. aureus* and *aap* in *S. epidermidis*.

Biofilms are notably prevalent in the middle ear mucosa of patients with COME, primarily produced by staphylococcal species. Researchers determined that 37.5% of the COME cases involve staphylococcal biofilm formation, potentially contributing to the chronic nature of the disease (Niedzielski, Chmielik & Stankiewicz, 2021). Our data indicate a higher prevalence of biofilm-related genes in *S. aureus* compared to CoNS (Table S3). Interestingly, a previous study revealed greater biofilm-forming capacity in *S. epidermidis* than *S. aureus*, likely enhanced by the unique presence of the *aap* gene, which when targeted by polyclonal antibodies can inhibit biofilm formation by up to 87% (Fey & Olson, 2010). This biofilm-forming capacity may be further facilitated by enhanced quorum sensing abilities in *S. epidermidis*, which is prevalent within middle ear mucosa. Notably, biofilms significantly enhance bacterial resistance to antibiotics by 10- to 1000-fold compared to planktonic bacterial forms and serve as reservoirs for genetic material exchange, including virulence and resistance genes, which augment the pathogenicity of staphylococci in COME (Sharma et al., 2023).

### Immune evasion genes

In our study, we characterized genes encoding virulence factors that facilitate immune evasion in *Staphylococcus* species. These factors include adenosine synthase A (*AdsA*), chemotaxis inhibitory protein (*CHIPS*), staphylococcal complement inhibitory protein (*SCIN*), immunoglobulin binding protein (*sbi*), and staphylococcal protein A (*spa*) (Table S4). Except for *CHIPS*, which is exclusive to the *S. aureus* strain NU84, these genes were universally present across the analyzed *S. aureus* strains. The unique presence of *CHIPS* in NU84 highlights its potential role in inhibiting neutrophil chemotaxis, suggesting a strain-specific adaptation for immune system evasion correspond with phylogenetic findings stated above (Bastian et al., 2018). *AdsA*, notably prevalent in CoNS, catalyzes the conversion of adenosine monophosphate to adenosine, exerting immunosuppressive effects such as macrophage inhibition, induction of regulatory T cells, and suppression of T cell activation (Leone & Emens, 2018). This finding underscores the sophisticated mechanisms *Staphylococcus* spp. deploy to modulate host immune responses.

Our analysis also identified the putative *capB* and *capC* genes predominantly in CoNS, but absent in *S. aureus*. These genes are responsible for the synthesis of polyglutamic acid capsules, atypical in staphylococci, which generally produce polysaccharide-based capsules (Sharma, Bhatnagar & Gaur, 2020). The presence of polyglutamic acid capsules, previously noted in *S. epidermidis* and *S. lugdunensis*, is significant (Da Silva Filho et al., 2020; Heilbronner & Foster, 2021). This difference in capsule composition not only suggests an evolutionary divergence between CoPS and CoNS but also points to a unique ecological niche adaptation, as evidenced by *S. epidermidis* surviving in high-salt environments (Fey & Olson, 2010).

### Enzyme-related genes

In our investigation of *Staphylococcus* strains, we identified 15 enzyme-encoding genes pivotal for pathogenicity, including cysteine protease (*sspB* & *sspC*), hyaluronate lyase (*hysA*), lipase (*geh* & *lip*), serine V8 protease (*sspA*), staphylocoagulase (*coa*), staphylokinase (*sak*), thermonuclease (*nuc*) and serine protease (*splA*, *splB*, *splC*, *splD*, *splE*, and *splF*) (Table S5). These genes are universally present in *S. aureus*, except in the NU84 strain which lacks the serine protease genes. Notably, serine proteases in *S. aureus* degrade host immunoglobulins, complement proteins, and cytokines, aiding in immune evasion and persistent infection establishment (Singh & Phukan, 2019; Paharik et al., 2016).

Within these enzymes, some of them are unique to *S. aureus* and not produced by CoNS. For instance, hyaluronate lyase which degrades hyaluronic acid and facilitates deeper tissue penetration is unique to *S. aureus* (Abdul Halim et al., 2020). Morever, staphylocoagulase is critical for differentiating CoPS from CoNS through its role in plasma coagulation, shielding bacteria from the immune response (Loof et al., 2015). Lastly, staphylokinase exclusive to CoPS, enhances tissue invasion and acts against specific antimicrobial peptides (Nguyen & Vogel, 2016). In contrast, CoNS prominently express thermonuclease and lipase, which disrupt neutrophil extracellular traps (NETs) and inhibit granulocyte phagocytic function respectively, supporting biofilm formation and chronic infection (Kenny et al., 2017).

### Toxin-related genes

Our genomic analysis of *Staphylococcus* strains also revealed 17 toxin-encoding genes, including (*hla*), beta hemolysin (*hlb*), delta hemolysin (*hld*), gamma hemolysin (*hlgA*, *hlgB*, *hlgC*), and a broad array of enterotoxins (*SEA* to *SEJ*), along with the toxic shock syndrome toxin (*TSST*) (Table S6). While *S. aureus* strains predominantly harbor these toxin genes, exceptions include TSST and several enterotoxins. Notably, *TSST*, typically associated with *S. aureus*, induces severe immune responses by triggering a cytokine storm, leading to toxic shock syndrome (Goda et al., 2021). However, recent evidence suggests that certain CoNS strains, like *S. epidermidis* and *S. simulans*, also produce TSST (Goda et al., 2021; Pomputius, Kilgore & Schlievert, 2023). Enterotoxins are superantigens discovered in *Staphylococcus* species which disrupt adaptive immune responses and are notorious for causing food poisoning (Cieza et al., 2024).

### Identification and characterization of genomic islands

Using IslandViewer, we predicted genomic islands (GIs) across the genomes of 16 *Staphylococcus* strains (Bertelli, Laird & Williams, 2017). Employing CD-HIT-EST, we clustered these GIs based on a threshold of 50% global sequence identity and alignment coverage, identifying 84 non-redundant GI clusters (Table S7).

Notably, Clusters 29 and 41 were exclusive to *S. epidermidis*. Cluster 29 contains the YycFG two-component system, crucial for stress adaptation and biofilm regulation which may contribute to bacterial persistence (Wu et al., 2019; Xu et al., 2017). Cluster 41 encompasses genes for cobalt-zinc-cadmium resistance, highlighting a potential heavy metal detoxification mechanism. Importantly, heavy metal co-resistance with antibiotics appear synergistic, as co-selective pressure from heavy metals promotes dissemination and persistence of antibiotic resistance genes in microbial communities (Gillieatt & Coleman, 2024). In *S. aureus*-specific Cluster 6, we identified a collection of virulence genes, including beta-hemolysin, staphylokinase, and others, potentially carried by the *ϕSa3int* prophage, known to enhance immune evasion capabilities (Nepal et al., 2025). Additionally, most CoNS displayed GIs in Clusters 27 and 80, containing staphylococcal pathogenicity islands (SaPIs) encoding superantigens. These elements provoke inflammatory responses through excessive cytokine release, exacerbating staphylococcal infection severity. SaPI mobility *via* helper phage facilitates horizontal gene transfer, promoting colonization and persistence in various host environments, leading to chronic infections (Novick, 2019). These findings highlight how genomic islands facilitate the acquisition of virulence and resistance traits support bacterial persistence and immune evasion in COME pathogenesis.

### Pan-genome analysis of *Staphylococcus* genomes

Our pan-genome analysis of these strains using Roary revealed 11,947 gene clusters, with only 655 genes constituting a conserved core genome, essential for basic bacterial functions and survival (Fig. S10). The remaining genes, forming the accessory genome, contribute to the phenotypic diversity and adaptability of the strains. Unique to CoPS (*S. aureus*), *sbn* operons, *plc*, and *patA* genes were identified, emphasizing distinct pathogenic mechanisms. The *sbn* operons are vital for iron acquisition from the host’s transferrin, significantly contributing to the virulence and persistence of *S. aureus* infections (Nakaminami et al., 2017). The *plc* gene encodes phosphoinositide-specific phospholipase C, which disrupts host cell membranes facilitating bacterial invasion and colonization (Nakamura et al., 2020). The *patA* gene contributes to immune evasion by modifying the peptidoglycan layer to resist lytic enzymes (Brott & Clarke, 2019). Although this protein has been found in many gram-positive bacteria, it is particularly prevalent in pathogenic species, providing them resistance against lysozyme (Sychantha et al., 2017). Altogether, our data suggests a higher pathogenic potential of *S. aureus* compared with CoNS due to the presence of many unique virulence related genes in *S. aureus*.

### Pan-genome analysis of *Staphylococcus aureus*

To elucidate the genomic architecture of *Staphylococcus aureus*, we performed a comprehensive pan-genome analysis using Roary on 18 genome sequences (three from this study and 15 from public databases), including clinical and environmental isolates (Table S2). This analysis identified 5,262 gene clusters, with 1,896 clusters (36%) constituting the core genome shared by all studied *S. aureus* strains. The remaining 3,366 clusters (64%) were classified as part of the accessory genome (Fig. S11).

The core genome predominantly encompasses genes essential for vital metabolic processes, including potassium metabolism, cell division, nitrogen metabolism, respiration, and phosphorus metabolism (Fig. S12). In contrast, genes involved in virulence, disease, and defense were primarily located within the accessory genome, indicating a potential for rapid acquisition or loss through horizontal gene transfer, facilitating adaptation to varying host environments and pathogenic challenges. For instance, one of the unique genes is *Bbp*, which encodes for bone sialoprotein-binding protein identified within the NU91 strain. This adhesive surface protein of *S. aureus* can bind to bone sialoprotein (BSP), a crucial component of dentine and bone extracellular matrix (Ao et al., 2017). The binding ability of bone sialoprotein binding protein to BSP in the ossicles of middle ear may contribute to enhanced biofilm formation and play a crucial role in the pathogenesis of *S. aureus*.

Unique to our *S. aureus* strains, we discovered several genes not found in strains from other research groups. These include *blaTEM-116* and *aph(3′)-IIa*, conferring resistance to beta-lactam and aminoglycoside antibiotics, respectively. Notably, these resistance genes were also identified in CoNS strains within our study, as evidenced by their presence in GI Cluster 0, suggesting a shared mechanism of resistance development among staphylococci. Another unique gene, *hgpA*, encodes the hemoglobin-haptoglobin-binding protein A, crucial for iron acquisition from host hemoglobin, highlighting its role in bacterial virulence and survival (Phillips et al., 2022).

### Pathogenicity prediction

Using the PathogenFinder tool, we assessed the potential pathogenicity of our strains. *S. aureus* exhibited the highest pathogenic potential, consistent with its array of virulence-related genes (Table S8) (Cosentino et al., 2013). This analysis underpins the role of genomic adaptations in the pathogenicity of *Staphylococcus* spp.

## Discussion

This study provides a comprehensive genomic analysis of *Staphylococcus* spp. isolated from paediatric COME patients in the UK. Through whole-genome sequencing, we have elucidated the phylogenetic relationships and identified novel genetic elements, including a potential new subspecies under *S. aureus* (NU84 strain), which necessitates further validation through experimental studies.

A significant finding of this study is the discovery of the *blaTEM-116* and *aph(3′)-IIa* resistance genes in all 16 *Staphylococcus* genomes isolated from COME patients. These genes, typically associated with Enterobacteriaceae, have not been previously reported in human *Staphylococcus* isolates. The *blaTEM-116* gene encodes an Extended Spectrum Beta-Lactamase (ESBL) that confers resistance to a broad range of beta-lactam antibiotics (Puah & Chua, 2019). While previous studies have reported the prevalence of the *blaTEM* gene in *S. aureus* isolates from patients with dental implants and children with oral and periodontal disease (Koukos et al., 2014; Sultana et al., 2022), these studies primarily refer to the *blaTEM-1a* variant, which is common in *S. aureus*. Notably, beta-lactamase *TEM-116* differs from beta-lactamase *TEM-1a* by two amino acid substitutions: valine to isoleucine at position 82 and alanine to valine at position 182 (UniProt). Therefore, this study presents the first report of the *blaTEM-116* gene in human *Staphylococcus* isolates. Similarly, the *aph(3′)-IIa* gene, which primarily inactivates neomycin and kanamycin, is commonly found in gram-negative bacteria. Although *aph(3′)-IIa* has been detected in *S. aureus*, its prevalence is extremely low (Woegerbauer et al., 2014). The presence of these resistance genes in all *Staphylococcus* strains raises concerns about the potential for horizontal gene transfer between gram-negative and gram-positive bacteria, highlighting the need for further research to understand the mechanisms of resistance gene acquisition and their clinical implications in COME treatment.

Our study uncovers novel insights into the antibiotic resistance mechanisms and pathogenic potential of *Staphylococcus* spp. isolated from paediatric COME patients. The identification of the *blaTEM-116* and *aph(3′)-IIa* genes, possibly acquired from the gram-negative plasmid pTAP6, represents an unusual instance of interspecies gene transfer, potentially facilitated by mechanisms not typically observed in gram-positive organisms (Dodsworth et al., 2010). This finding raises concerns about the emergence of *Staphylococcus* strains with enhanced pathogenicity due to novel resistance capabilities.

The high prevalence of *blaZ* and *blaTEM-116* genes in our *Staphylococcus* strains may impact the effectiveness of commonly used antibiotics, such as amoxicillin, trimethoprim-sulfamethoxazole, and augmentin (amoxicillin + clavulanic acid) (Venekamp et al., 2016). Previous studies have demonstrated that planktonic *S. aureus* with high resistance to amoxicillin but lower resistance to amoxicillin with clavulanic acid (Sabino et al., 2022). Interestingly, biofilm-forming *S. aureus* has shown increased tolerance to both amoxicillin (100%) and amoxicillin with clavulanic acid (60.9%) (Sabino et al., 2022). This tolerance mechanism is attributed to the formation of biofilm, which blocks penetration of antibiotics and prevents their effective action against *S. aureus*. The synergistic effect of clavulanic acid on amoxicillin is achieved by its inhibitory action against beta-lactamases, thereby extending the antibacterial activity of amoxicillin against beta-lactamases producing bacteria (Bush & Bradford, 2016). Planktonic *S. aureus* resistant to amoxicillin with clavulanic acid are believed to possess the *mecA* gene because the presence of clavulanic acid as beta-lactamase inhibitors can be overcome by lower affinity of penicillin-binding protein 2a (PBP2a) with beta-lactam antibiotics. This explanation is further supported by another study showing higher resistance of methicillin-resistant S. aureus (80%) to amoxicillin with clavulanic acid compared to methicillin-sensitive S. aureus (62%) (Idrees et al., 2023).

In term of virulence, comparative virulence factor analysis revealed that CoPS possess various virulence genes, indicating high virulence potential in causing COME onset, while CoNS harbor genes promoting persistence, such as biofilm formation and immune evasion, contributing to the chronic nature of COME. Controlling the rapid dissemination of *blaZ*, *blaTEM-116*, *mecA* resistance genes, and biofilm-related genes among staphylococci may be crucial in preventing COME from causing permanent conductive hearing loss in the young generation. The identification of these resistance determinants highlights the importance of routine genomic surveillance in otitis media cases to guide targeted antibiotic prescribing and reduce inappropriate COME treatment. Future research should explore novel therapeutic approaches like biofilm-discrupting agents and better understand resistance gene acquisition mechanisms.

However, several limitations should be acknowledged when interpreting the findings of this genomic analysis of COME-associated *Staphylococcus* strains. The relatively small sample size (16 strains) and geographical restriction to UK patients limit generalizability. Reliance on draft assemblies rather than fully closed genomes may reduce resolution of genomic elements. All findings are based on *in silico* predictions without phenotypic validation through antimicrobial susceptibility testing or gene expression profiling. The absence of comparative analysis with other bacterial pathogen or healthy controls could restrict understanding of *Staphylococcus-* specific contributions to COME pathogenesis. Moreover, sequencing artifacts like chimeric reads may cause false positives, while low coverage could result in false negatives (Qin et al., 2023; Kong et al., 2018).

In overall, this study provides valuable insight into the genomic diversity, antibiotic resistance, and virulence potential of *Staphylococcus* spp. in paediatric COME patients, emphasizing the need for continued surveillance and the development of effective strategies to combat the emergence of multidrug-resistant strains.

## Conclusions

Our genomic analyses reveal that *Staphylococcus* spp. possess significant pathogenic potential to contribute to COME, primarily through the acquisition of resistance and virulence genes. The transmission of antibiotic resistance genes from gram-negative to gram-positive bacteria, as evidenced in our study, marks a critical evolutionary adaptation that could enhance the pathogenicity of *Staphylococcus* strains. This study not only advances our understanding of the genetic foundations of COME-associated *Staphylococcus* spp. but also highlights the urgent need for novel therapeutic strategies to combat the rising challenge of antibiotic resistance in these clinically significant pathogens. These discoveries are pivotal for rethinking current treatment strategies for COME, suggesting a move towards more personalized antimicrobial therapies that are tailored to the specific resistance profiles of bacterial strains.

## Supplemental Information

10.7717/peerj.20782/supp-1 Supplemental Information 1 Sequencing reads of 16 Staphylococcus strains

10.7717/peerj.20782/supp-2 Supplemental Information 2 Information of additional Staphylococcus strains used in this study

10.7717/peerj.20782/supp-3 Supplemental Information 3 Distribution of biofilm-related genes within the genomes of CoPS and CoNS ”1”= presence of gene; ”0”= absence of gene

10.7717/peerj.20782/supp-4 Supplemental Information 4 Distribution of virulence factors involved in immune evasion within the genomes of CoPS and CoNS ”1”= presence of gene; ”0”= absence of gene

10.7717/peerj.20782/supp-5 Supplemental Information 5 Distribution of enzymes that potentially act as virulence factors within the genomes of CoPS and CoNS ”1”= presence of gene; ”0”= absence of gene

10.7717/peerj.20782/supp-6 Supplemental Information 6 Distribution of toxins that act as virulence factors within the genomes of CoPS and CoNS ”1”= presence of gene; ”0”= absence of gene

10.7717/peerj.20782/supp-7 Supplemental Information 7 Putative Genomic Island clusters Puresence of genomic island is indicated in green color while absence of genomic island is indicated in red color

10.7717/peerj.20782/supp-8 Supplemental Information 8 PathogenFinder analysis of Staphylococcus strains

10.7717/peerj.20782/supp-9 Supplemental Information 9 The 16S rRNA-based phylogenetic tree depicting the relationship between Staphylococcus species The tree was constructed by using the maximum-likelihood method. In total, 1000 bootstrap replicates were used. Branch length indicates divergence and bootstrap support values are also shown.

10.7717/peerj.20782/supp-10 Supplemental Information 10 The multiple gene-based phylogenetic tree depicting the relationship between Staphylococcus species The tree was constructed by using the maximum-likelihood method. In total, 1000 bootstrap replicates were used. Branch length indicates divergence and bootstrap support values are also shown.

10.7717/peerj.20782/supp-11 Supplemental Information 11 The core-genome SNP-based phylogenetic tree depicting the relationship between Staphylococcus species The tree was inferred by using the Neighbour-joining method. In total, 1000 bootstrap replicates were used. Branch length indicates divergence and bootstrap support values are also shown.

10.7717/peerj.20782/supp-12 Supplemental Information 12 ANIb analysis of Staphylococcus species * Staphylococcus* strains showing ANIb value above 95% which belong to the same species were clustered together and visualized in deep blue color through heatmap.

10.7717/peerj.20782/supp-13 Supplemental Information 13 GGDC analysis of Staphylococcus species * Staphylococcus* strains showing dDDH above 70% which belong to the same species were clustered together and visualized in deep blue color through heatmap.

10.7717/peerj.20782/supp-14 Supplemental Information 14 GGDC analysis of closely related subspecies within Staphylococcus hominis Pairwise genome comparison was performed on strain NU72 and other published *Staphylococcus hominis* strains including CCUG 42399, MBBF12-19J, K1, and NCTC11320, C80 from NCBI database and R22, DM122, KL243 from American Type Culture Collection.

10.7717/peerj.20782/supp-15 Supplemental Information 15 The core-genome SNP-based phylogenetic tree constructed using MEGA11 for Staphylococcus hominis strains Our analysis showed two distinct clades which represent *S. hominis subsp. hominis* and *S. hominis subsp. novobiosepticus*.

10.7717/peerj.20782/supp-16 Supplemental Information 16 Heatmap constructed using the GGDC result of closely related subspecies within Staphylococcus aureus Pairwise genome comparisons were performed on strains NU84, NU91, and NU65 and the genome sequences of published *S. aureus* strains including ST1464, MVF-7, 2395 USA500, CFSAN007835, UP_490, JASL01, UP_1452, and NCTC13811 from NCBI database.

10.7717/peerj.20782/supp-17 Supplemental Information 17 Putative resistance genes identified in the assembled genomes of Staphylococcus species Deep blue color= presence of a particular resistance gene; blank color= absence of resistance gene.

10.7717/peerj.20782/supp-18 Supplemental Information 18 Pan genome analysis of 16 Staphylococcus strains using Roary Staphylococcal genome were subdivided into two groups: core genome and accessory genome.

10.7717/peerj.20782/supp-19 Supplemental Information 19 Pan genome analysis of 18 Staphylococcus aureus using Roary Staphylococcal genome were subdivided into two groups: core genome and accessory genome.

10.7717/peerj.20782/supp-20 Supplemental Information 20 Functional annotation of Staphylococcus aureus gene within core genome and accessory genomes using RAST These genes are distributed into different functional categories: A is cofactors, vitamins, prosthetic groups, pigments; B is cell wall and capsule; C is virulence, disease and defense; D is potassium metabolism; E is potassium metabolism; F is membrane transport; G is iron acquisition and metabolism; H is RNA metabolism; I is nucleosides and nucleotides, J is protein metabolism; K is cell division; L is regulation and cell signaling; M is DNA metabolism; N is fatty acids, lipids and isoprenoids, O is nitrogen metabolism, P is dormancy and sporulation; Q is respiration; R is stress response; S is metabolism of aromatic compounds; T is amino acids and derivatives; U is sulfur metabolism; W is phosphorus metabolism; W is carbohydrates. The numbers indicate the gene number corresponding to each category.

## Additional Information and Declarations

### DNA Deposition

The following information was supplied regarding the deposition of DNA sequences: The sequence data is available at NCBI Genbank: PRJNA722783 with genome accessions JAGSWU000000000, JAGSWV000000000, JAGSWW000000000, JAGSWX000000000, JAGSWY000000000, JAGSWZ000000000, JAGSWQ000000000, JAGSWR000000000, JAGSWS000000000, JAGSWT000000000, JAGSXB000000000, JAGSXC000000000, JAGSXD000000000, JAGSXE000000000, JAGSXF000000000, JAGSXG000000000, and JAGSXA000000000.

### Data Availability

The following information was supplied regarding data availability: The raw data are available in the Supplemental Files.
