# Supplementary material for: Genomic insights into the diversity, antibiotic resistance, and virulence potential of staphylococci isolated from pediatric patients with chronic otitis media with effusion (COME)
Source: PeerJ. 2026 Mar 24;14:e20782. doi: 10.7717/peerj.20782 (PMC13024242; doi:10.7717/peerj.20782)
Supplement: Supplemental Information 9 — The tree was constructed by using the maximum-likelihood method. In total, 1000 bootstrap replicates were used. Branch length indicates divergence and bootstrap support values are also shown. [file peerj-14-20782-s009.pdf]

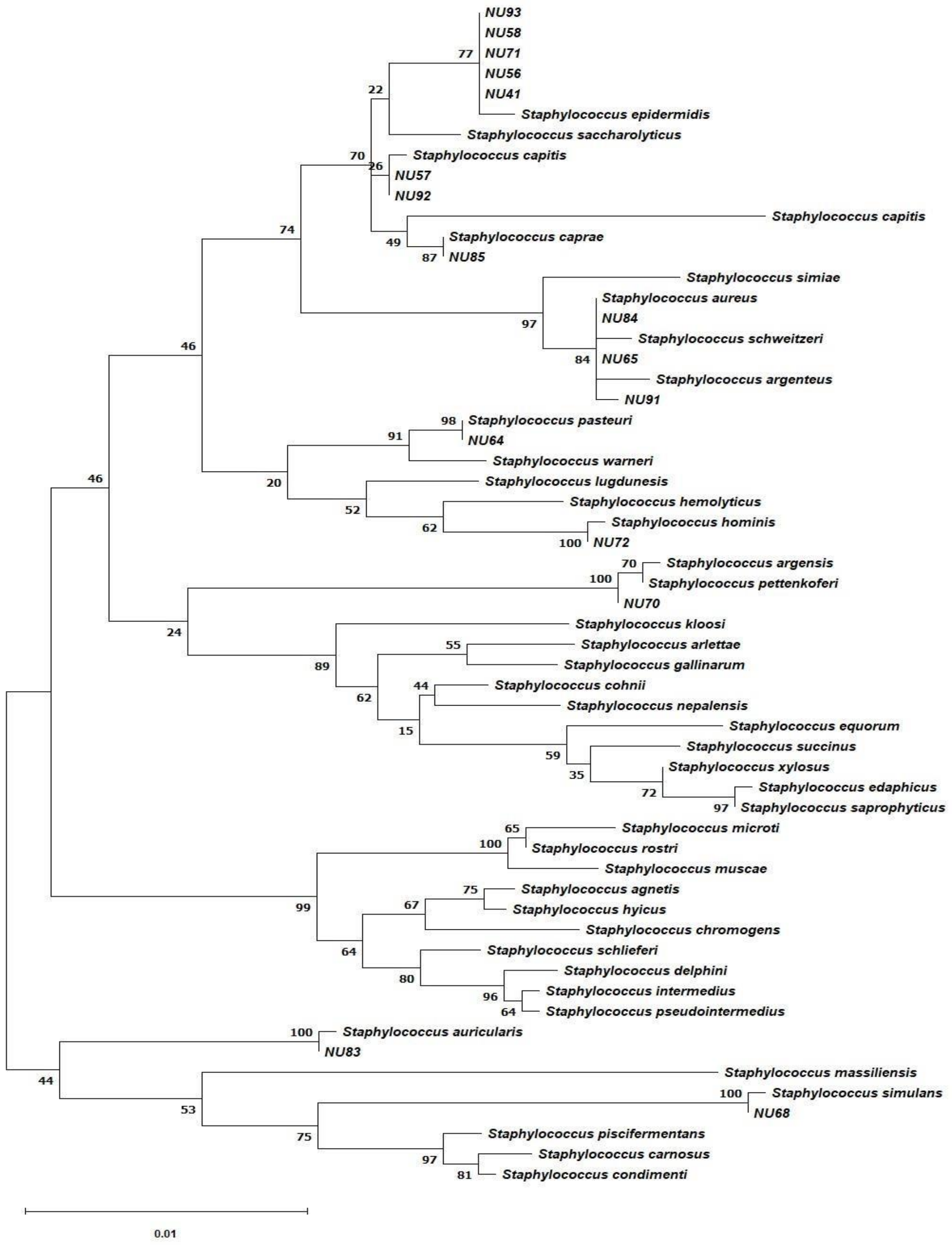

Figure S1: The 16S rRNA-based phylogenetic tree depicting the relationship between *Staphylococcus* species. The tree was constructed by using the maximum-likelihood method. In total, 1000 bootstrap replicates were used. Branch length indicates divergence and bootstrap support values are also shown.
