# Supplementary material for: Genomic insights into the diversity, antibiotic resistance, and virulence potential of staphylococci isolated from pediatric patients with chronic otitis media with effusion (COME)
Source: PeerJ. 2026 Mar 24;14:e20782. doi: 10.7717/peerj.20782 (PMC13024242; doi:10.7717/peerj.20782)
Supplement: Supplemental Information 12 — Staphylococcus strains showing ANIb value above 95% which belong to the same species were clustered together and visualized in deep blue color through heatmap. [file peerj-14-20782-s012.pdf]

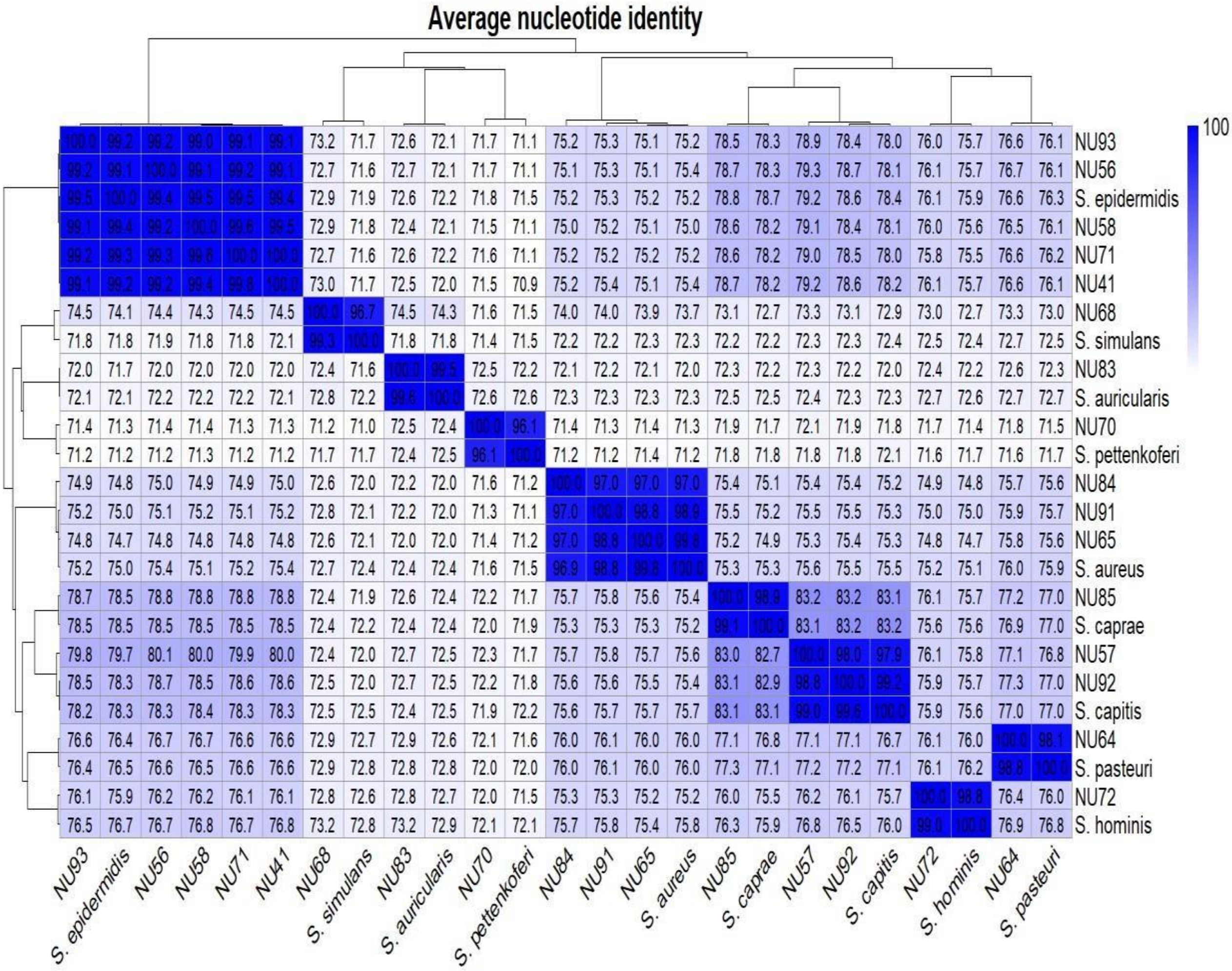

Figure S4: ANIb analysis of *Staphylococcus* species. *Staphylococcus* strains showing ANIb value above 95% which belong to the same species were clustered together and visualized in deep blue color through heatmap.
