# Supplementary material for: Genomic insights into the diversity, antibiotic resistance, and virulence potential of staphylococci isolated from pediatric patients with chronic otitis media with effusion (COME)
Source: PeerJ. 2026 Mar 24;14:e20782. doi: 10.7717/peerj.20782 (PMC13024242; doi:10.7717/peerj.20782)
Supplement: Supplemental Information 14 — Pairwise genome comparison was performed on strain NU72 and other published Staphylococcus hominis strains including CCUG 42399, MBBF12-19J, K1, and NCTC11320, C80 from NCBI database and R22, DM122, KL243 from American Type Culture Collection. [file peerj-14-20782-s014.pdf]

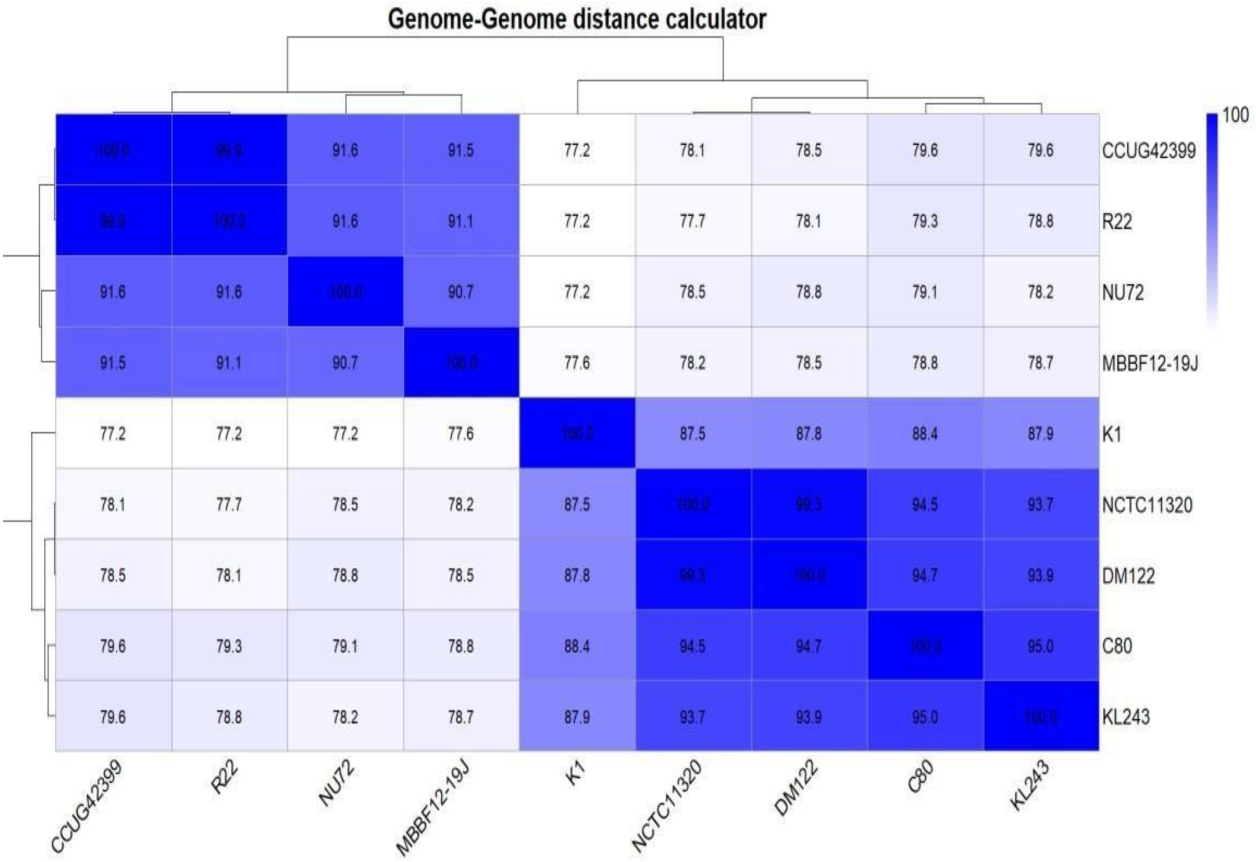

Figure S6: GGDC analysis of closely related subspecies within *Staphylococcus hominis*.

Pairwise genome comparison was performed on strain NU72 and other published *Staphylococcus hominis* strains including CCUG 42399, MBBF12-19J, K1, and NCTC11320, C80 from NCBI database and R22, DM122, KL243 from American Type Culture Collection.
