# Supplementary material for: Genomic insights into the diversity, antibiotic resistance, and virulence potential of staphylococci isolated from pediatric patients with chronic otitis media with effusion (COME)
Source: PeerJ. 2026 Mar 24;14:e20782. doi: 10.7717/peerj.20782 (PMC13024242; doi:10.7717/peerj.20782)
Supplement: Supplemental Information 15 — Our analysis showed two distinct clades which represent S. hominis subsp. hominis and S. hominis subsp. novobiosepticus. [file peerj-14-20782-s015.pdf]

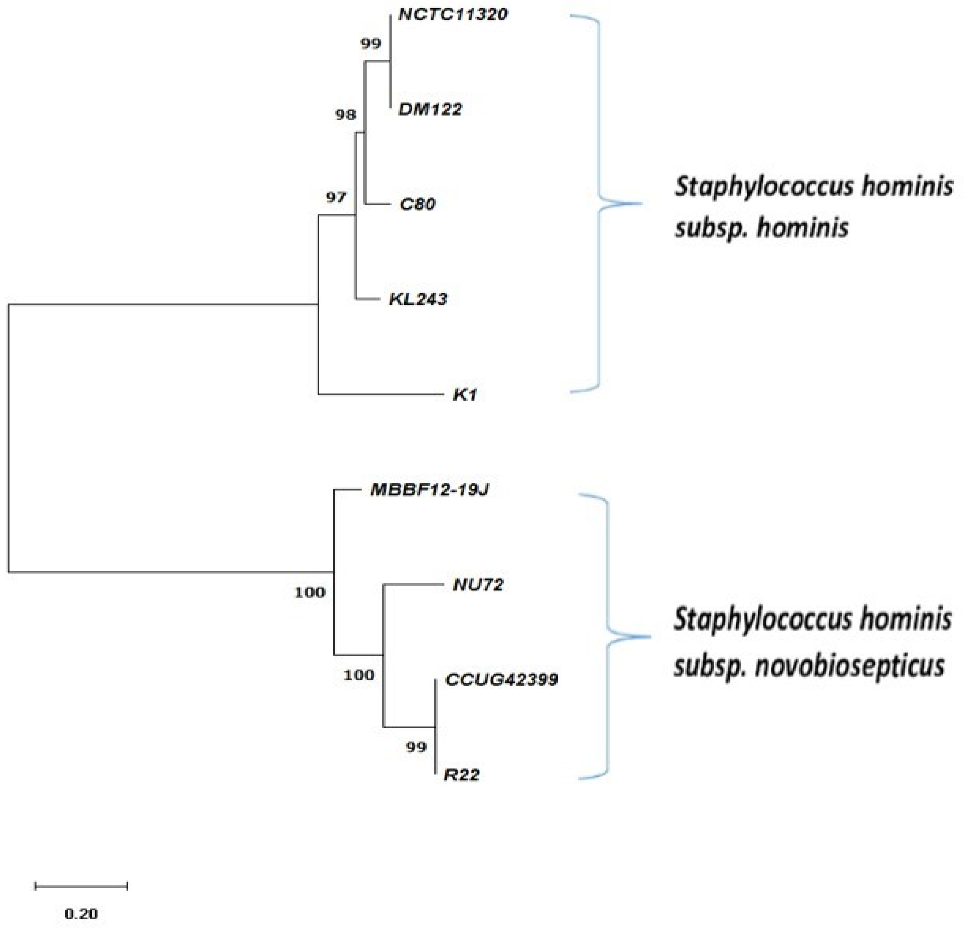

Figure S7: The core-genome SNP-based phylogenetic tree constructed using MEGA11 for *Staphylococcus hominis* strains. Our analysis showed two distinct clades which represent *S. hominis subsp. hominis* and *S. hominis subsp. novobiosepticus*.
