# Supplementary material for: Genomic insights into the diversity, antibiotic resistance, and virulence potential of staphylococci isolated from pediatric patients with chronic otitis media with effusion (COME)
Source: PeerJ. 2026 Mar 24;14:e20782. doi: 10.7717/peerj.20782 (PMC13024242; doi:10.7717/peerj.20782)
Supplement: Supplemental Information 16 — Pairwise genome comparisons were performed on strains NU84, NU91, and NU65 and the genome sequences of published S. aureus strains including ST1464, MVF-7, 2395 USA500, CFSAN007835, UP_490, JASL01, UP_1452, and NCTC13811 from NCBI database. [file peerj-14-20782-s016.pdf]

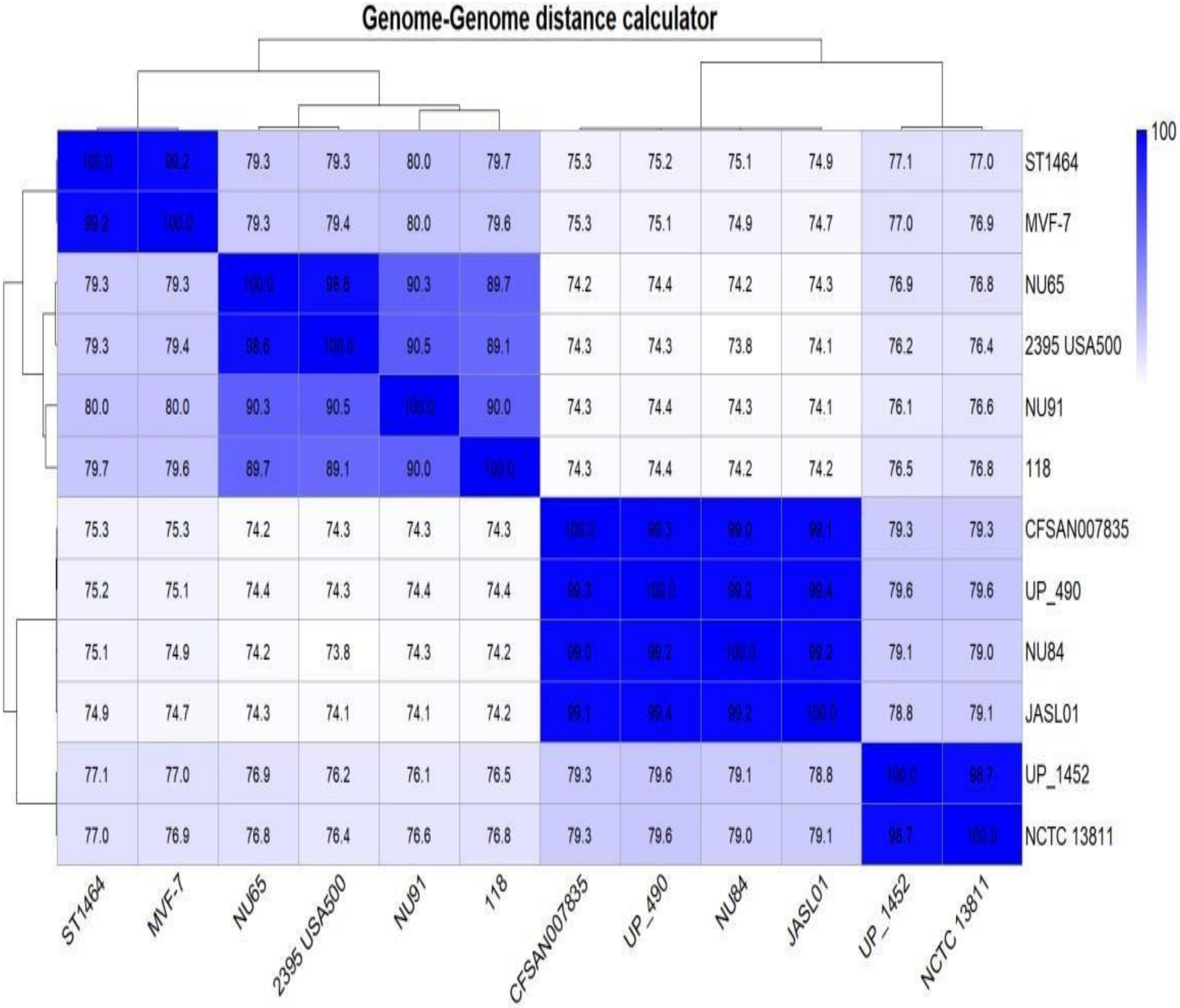

Figure S8: Heatmap constructed using the GGDC result of closely related subspecies within *Staphylococcus aureus*. Pairwise genome comparisons were performed on strains NU84, NU91, and NU65 and the genome sequences of published *S. aureus* strains including ST1464, MVF-7, 2395 USA500, CFSAN007835, UP_490, JASL01, UP_1452, and NCTC13811 from NCBI database.
