# Supplementary material for: Genomic insights into the diversity, antibiotic resistance, and virulence potential of staphylococci isolated from pediatric patients with chronic otitis media with effusion (COME)
Source: PeerJ. 2026 Mar 24;14:e20782. doi: 10.7717/peerj.20782 (PMC13024242; doi:10.7717/peerj.20782)
Supplement: Supplemental Information 17 — Deep blue color= presence of a particular resistance gene; blank color= absence of resistance gene. [file peerj-14-20782-s017.pdf]

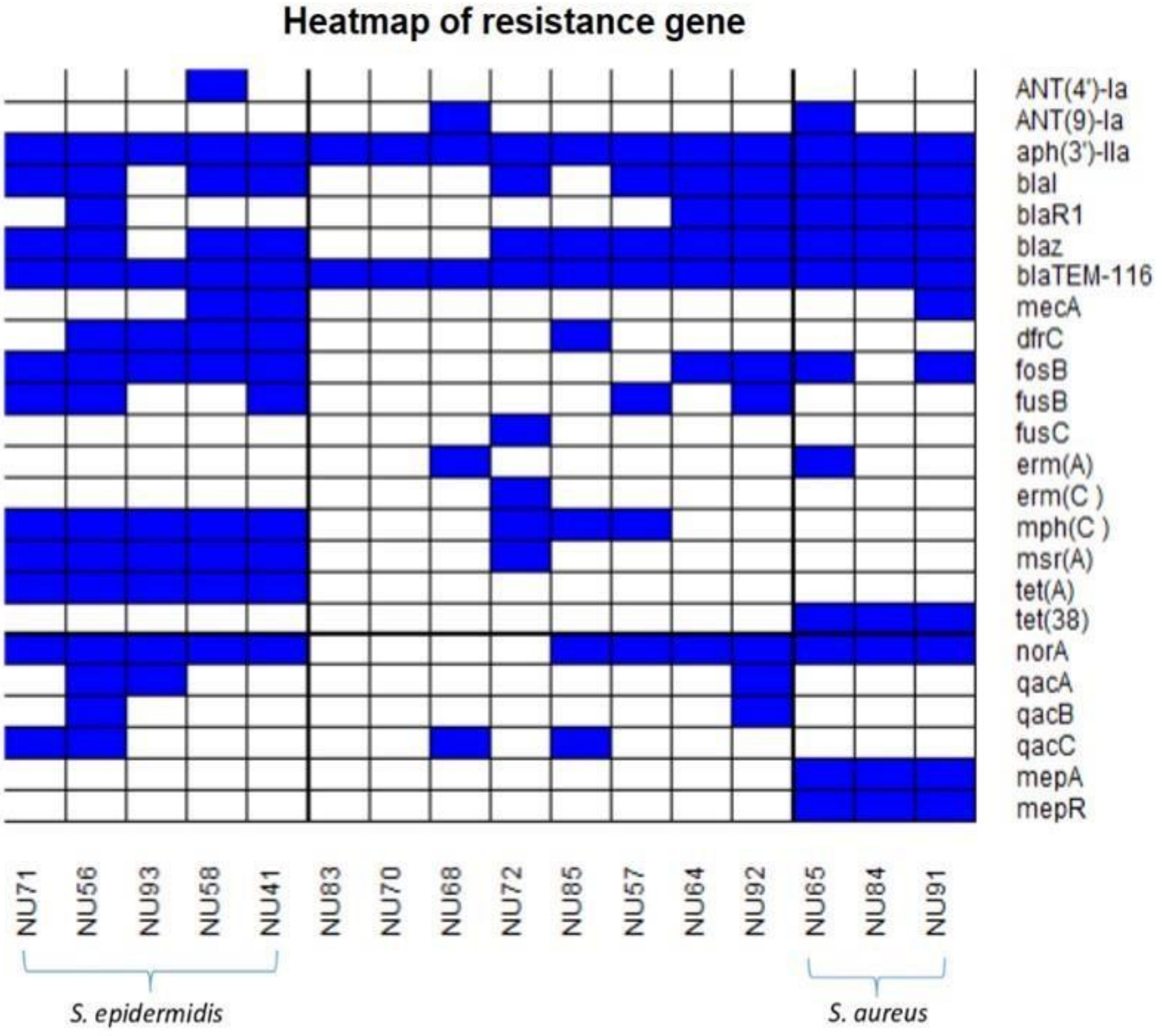

Figure S9: Putative resistance genes identified in the assembled genomes of *Staphylococcus* spp. Deep blue color= presence of a particular resistance gene; blank color= absence of resistance gene.
