# Supplementary material for: Genomic insights into the diversity, antibiotic resistance, and virulence potential of staphylococci isolated from pediatric patients with chronic otitis media with effusion (COME)
Source: PeerJ. 2026 Mar 24;14:e20782. doi: 10.7717/peerj.20782 (PMC13024242; doi:10.7717/peerj.20782)
Supplement: Supplemental Information 19 — Staphylococcal genome were subdivided into two groups: core genome and accessory genome. [file peerj-14-20782-s019.pdf]

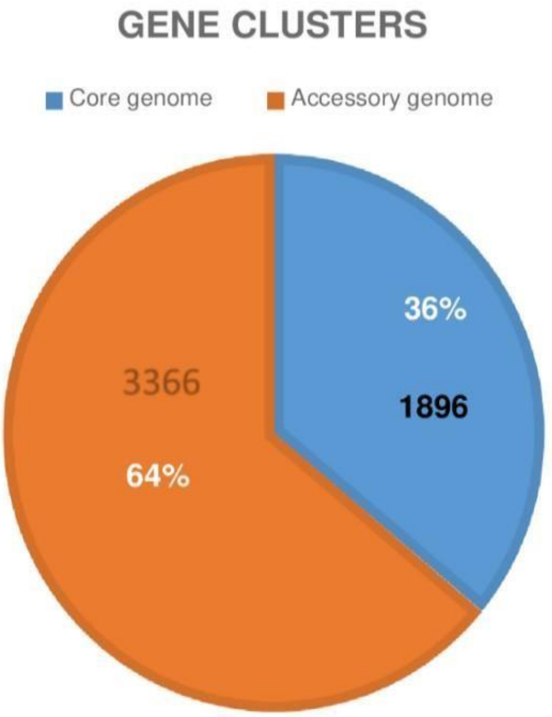

Figure S11: Pan genome analysis of 18 *Staphylococcus aureus* using Roary. Staphylococcal genome were subdivided into two groups: core genome and accessory genome.
