# Supplementary material for: Genomic insights into the diversity, antibiotic resistance, and virulence potential of staphylococci isolated from pediatric patients with chronic otitis media with effusion (COME)
Source: PeerJ. 2026 Mar 24;14:e20782. doi: 10.7717/peerj.20782 (PMC13024242; doi:10.7717/peerj.20782)
Supplement: Supplemental Information 20 — These genes are distributed into different functional categories: A is cofactors, vitamins, prosthetic groups, pigments; B is cell wall and capsule; C is virulence, disease and defense; D is potassium metabolism; E is potassium metabolism; F is membrane transport; G is iron acquisition and metabolism; H is RNA metabolism; I is nucleosides and nucleotides, J is protein metabolism; K is cell division; L is regulation and cell signaling; M is DNA metabolism; N is fatty acids, lipids and isoprenoids, O is nitrogen metabolism, P is dormancy and sporulation; Q is respiration; R is stress response; S is metabolism of aromatic compounds; T is amino acids and derivatives; U is sulfur metabolism; W is phosphorus metabolism; W is carbohydrates. The numbers indicate the gene number corresponding to each category. [file peerj-14-20782-s020.pdf]

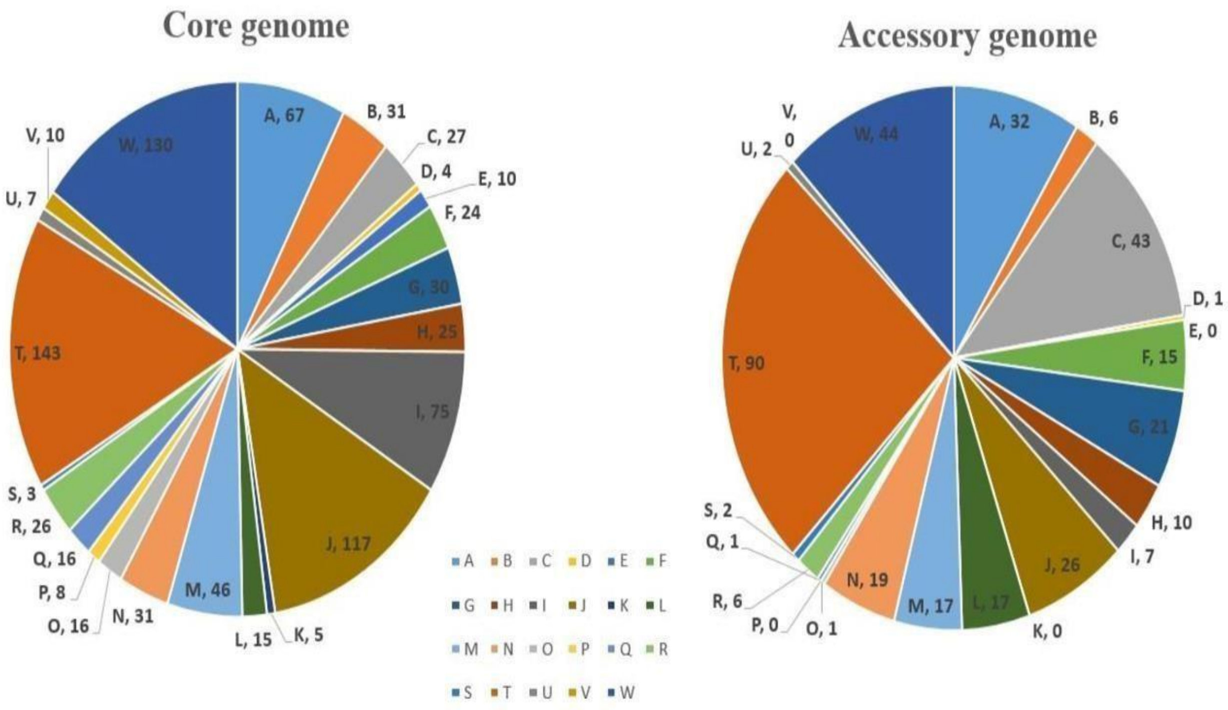

Figure S12: Functional annotation of *Staphylococcus aureus* gene within core genome and accessory genome using RAST. These genes are distributed into different functional categories: A is cofactors, vitamins, prosthetic groups, pigments; B is cell wall and capsule; C is virulence, disease and defense; D is potassium metabolism; E is potassium metabolism; F is membrane transport; G is iron acquisition and metabolism; H is RNA metabolism; I is nucleosides and nucleotides, J is protein metabolism; K is cell division; L is regulation and cell signaling; M is DNA metabolism; N is fatty acids, lipids and isoprenoids, O is nitrogen metabolism, P is dormancy and sporulation; Q is respiration; R is stress response; S is metabolism of aromatic compounds; T is amino acids and derivatives; U is sulfur metabolism; W is phosphorus metabolism; W is carbohydrates. The numbers indicate the gene number corresponding to each category.
